# 563. Experience of a Private Hospital in the Treatment of COVID-19 Pneumonia in Veracruz, Mexico

**DOI:** 10.1093/ofid/ofab466.761

**Published:** 2021-12-04

**Authors:** Luis Del Carpio-Orantes, Luis Alberto Márquez-Rodriguez, José Luis García-Pérez, Christian Alberto Rodríguez-Santos, Carlos Yahir Llaven-Velázquez, Eduardo Gutiérrez-Martínez, Anel Concepción Lucho-Morales, Juan Carlos Mendoza-Hernández, Jorge José García-Ortíz, Luis Jaime Medrano-Rios, Enrique Ruiz-Castro, Manuel Alejandro Ortiz-Hernández

**Affiliations:** 1 Instituto Mexicano del Seguro Social; Sociedad Mexicana de Virología, Veracruz, Veracruz-Llave, Mexico; 2 Hospital D′María, Veracruz, Veracruz-Llave, Mexico

## Abstract

**Background:**

Large mortality rates have been reported in the Mexican public health system, however in the experiences of private hospitals that have resources and infrastructure this is lower compared to the national average.

**Methods:**

Descriptive and retrospective study. Adult patients treated for pneumonia due COVID-19 from April to December 2020 are entered into the study. Its general characteristics such as gender and age, comorbidities, influenza vaccination history, clinical characterization, laboratory and tomographic diagnosis of sars cov2 pneumonia are studied, as well as the drug and oxygen therapy treatments received and finally, its evolution and clinical outcome.

**Results:**

132 patients were studied, of which 51% were female. The main age groups affected were 65 and over (43.9%), 50-59 years (20.4%) and 25-44 years (16.6%). The main comorbidities found were: arterial hypertension (43.9%), Diabetes mellitus 2 (33.3%), heart disease (11.3%) and obesity (10.6%). 95.4% of the patients were not vaccinated against influenza. The main symptoms reported were: fever (92%), cough (87%), dyspnea (76%) and headache (52%). The diagnosis was confirmed with RT-PCR in 63%, reporting negative RT-PCR in 36%; the antigen test was positive in 1%. Regarding the findings of the chest computed tomography, CORADS 5 was reported in 30%, CORADS 6 in 3% and CORADS 4 in 20%. The main treatments used in patients with severe inflammatory pneumonia were: steroids (98%), enoxaparin (100%), tocilizumab (20%), baricitinib (60%), direct oral anticoagulants (10%), fibroquel (5%). 60% were treated with a combination of two or more drugs. The main oxygenation contributions were: 20% nasal tips - mask/reservoir, 60% high flow nasal cannula, 20% mechanical ventilation. In 95% the prone position was indicated. Regarding the clinical evolution, 65.1% were towards improvement, 17.4% died, 12.1% requested transfer to another unit and 5.3% requested voluntary discharge. Overall mortality was 17%.

Medications in ICU

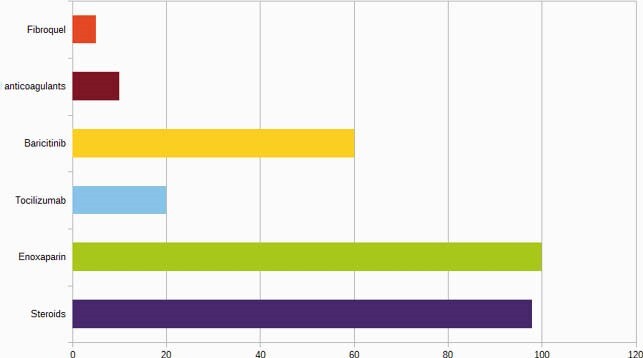

Ventilation strategies

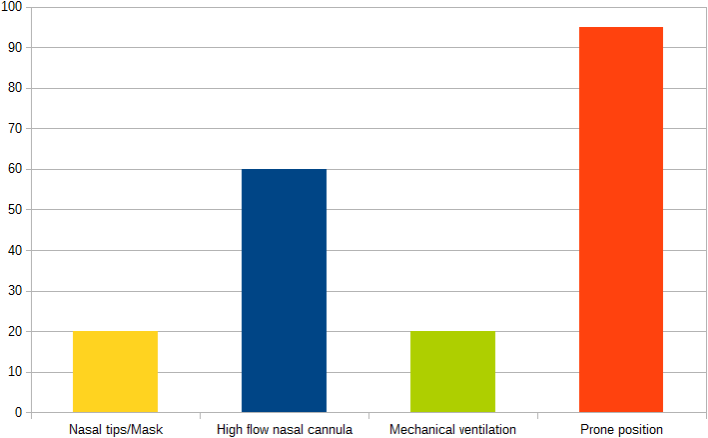

**Conclusion:**

A hospital strategy that has the necessary resources and infrastructure as well as openness to the use of medication with emergency approvals for its use or off-label indications, can help limit morbidity and mortality in vulnerable populations and manifest risk factors such as Mexican population

**Disclosures:**

**All Authors**: No reported disclosures

